# Xolair® (omalizumab) enrollment in a tertiary care allergy and asthma clinic in Canada

**DOI:** 10.1186/1710-1492-10-S2-A5

**Published:** 2014-12-18

**Authors:** Jodi Cameron, Jennifer Forgie, Alicia Ring, Stephanie Santucci, Caroline Rizk, Hoang Pham, John O’Quinn, William H Yang

**Affiliations:** 1 Allergy and Asthma Research Centre, Ottawa, ON, Canada; 2 Department of Clinical Immunology and Allergy, McGill University, Montreal, Canada; 3 University of Ottawa Medical School, Ottawa, ON, Canada

## Background

Xolair ^®^ (omalizumab) has been approved in Canada since 2004 for the treatment of moderate to severe persistent allergic asthma in patients 12 years of age. The use of omalizumab in severe persistent allergic asthma may lead to decrease health care utilization through emergency room (ER) visits, hospitalizations, visits to health care providers, as well as, decrease the use of corticosteroids and improve the overall quality of life (QoL).

## Methods

Data collected from patient enrollment and QoL questionnaires completed at specific intervals during treatment with omalizumab at our large tertiary care clinic from 2004 to 2014 was analyzed.

## Results

A steady number of patients were enrolled each year since 2004, showing its greatest increase in enrollment numbers since 2012. Our data indicates that the majority of patients improved with significantly less asthma exacerbation, less ER visits and hospitalizations, less use of inhaled and oral corticosteroids and better QoL.

## Conclusion

Omalizumab is effective in the treatment of moderate and severe allergic asthma. It improves QoL and reduces asthma exacerbations, ER visits and hospitalizations, and use of inhaled and oral corticosteroids.

